# Prevalence of Antibiotic Purchase Online and Associated Factors Among Chinese Residents: A Nationwide Community Survey of 2019

**DOI:** 10.3389/fphar.2021.761086

**Published:** 2021-11-03

**Authors:** Na Sun, Yanhong Gong, Jiaming Liu, Jianxiong Wu, Rongrong An, Yue Dong, Yi Zhu, Ketao Mu, Guopeng Zhang, Xiaoxv Yin

**Affiliations:** ^1^ Department of Social Medicine and Health Management, School of Public Health, Tongji Medical College, Huazhong University of Science and Technology, Wuhan, China; ^2^ Department of Radiology, Tongji Hospital, Tongji Medical College, Huazhong University of Science and Technology, Wuhan, China; ^3^ Department of Nuclear Medicine, Tongji Hospital, Tongji Medical College, Huazhong University of Science and Technology, Wuhan, China

**Keywords:** antibiotic, online purchase, behavior, national prevalence, associated factor

## Abstract

**Introduction:** Online sales of antibiotics have increased public access to these medicines. This study aimed to analyze the online antibiotic purchase behavior of the Chinese residents and identify its associated factors.

**Methods:** We conducted a nationwide cross-sectional online survey among Chinese community residents from January 20 to February 28, 2019. A structured questionnaire was used to collect data on their sociodemographic characteristics, health-related variables, and the online antibiotic purchase behavior in the past 3 months. Descriptive statistics and logistic regression analyses were used. The statistical analyses were performed using SAS version 9.4 (SAS Institute Inc.).

**Results:** A total of 101,120 respondents were included in the analysis. The weighted prevalence of antibiotic purchase online was 3.71% (95% CI, 3.53–3.88%). Residents who purchased antibiotics online were more likely to be older (age≥65 years), be a male, live in rural areas, have a higher education level, report an excellent economic status, suffer from chronic diseases, and search for health information on the internet.

**Conclusion:** Numerous residents had purchased antibiotics online in the past 3 months throughout China. We should pay more attention to this behavior. There is a need to strengthen regulation of antibiotic sales online and improve public education on antibiotic purchase online. More comprehensive information on antibiotic purchase online as well as the advantages and disadvantages of online sales of antibiotics should be investigated in the future studies.

## Introduction

Irrational use of antibiotics and the antibiotic resistance it has caused pose a serious threat to globe health (Weber, 2005; Laxminarayan et al., 2013; Zaman et al., 2017). The irrational use of antibiotics among residents consists mainly of the over-the-counter antibiotic purchases, storage and use. Throughout the world, the prevalence of purchasing antibiotics without a prescription, storage of antibiotics, or self-medication with antibiotics was high among residents. For example, 25.5 and 64.2% of the residents had purchased antibiotics without a prescription in Italy and China, respectively (Bianco et al., 2020; Lin et al., 2020); the prevalence of antibiotic storage among the residents in the United States and Australian Chinese migrants reached 48 and 47%, respectively (Hu and Wang, 2014; Grigoryan et al., 2019); and the overall prevalence of antimicrobial self-medication was 38.8% in low-and-middle-income countries (Ocan et al., 2015).

With the development of electronic commerce, online sales of antibiotics have increased public access to these medicines, which is an enormous challenge for antibiotic stewardship (Mainous et al., 2009; Orizio et al., 2011; Boyd et al., 2017). As a new platform for purchasing medicines, the online pharmacy was not properly supervised, and the over-the-counter antibiotic sales were common, which could pose a threat to the original system of antibiotic stewardship. For example, researches in the United Kingdom and the United States reported that 45.0 and 36.2% of online pharmacies dispensed antibiotics without a prescription, respectively (Mainous et al., 2009; Boyd et al., 2017). Furthermore, online pharmacy staff provided clients with limited information on the antibiotic use due to the lack of face-to-face communication (Gong et al., 2020). This may increase the risk of irrational use of antibiotics among residents, such as shortening the course of medication and changing the therapeutic dose, and therefore, result in antibiotic resistance. Facing the aforementioned challenges, there is a need to analyze the online antibiotic sale and purchase behavior. However, current researches mainly focused on the antibiotic sale behavior of online pharmacies that was a supply side of medicines, such as their over-the-counter antibiotic sales and service quality, and ignored the antibiotic purchase behavior of residents who was the demand side of medicines. Therefore, it is necessary to explore the purchases of antibiotics among residents, so as to attract administrator’s attention to this behavior, as well as provide evidence for antibiotic stewardship.

China is one of the countries with a serious problem of irrational use of antibiotics (Fang, 2014; Lv et al., 2014; Yu et al., 2014). In addition, the electronic commerce has developed rapidly and internet sales of medicines have increased substantially in this country. Total drug sales on the internet in China increased by 29%; from US$ 1.1 billion in 2017 to US$ 1.5 billion in 2018 (Beijing Weiming Penguin, 2019). Previous studies showed that, online sales of prescription-only medicines were not well regulated in China, and 67.8% of online pharmacies sold antibiotics without a prescription (Gong et al., 2020), which might encourage Chinese residents to use an online platform for antibiotic purchase. In view of these circumstances, we conducted this study aiming to analyze the online antibiotic purchase behavior of Chinese residents and identify its associated factors. As the first research report on the prevalence of antibiotic purchase online among residents and its associated factors, this study was hoped to help the researchers and policymakers worldwide better understand the online antibiotic consumption behavior of the general population, as well as provide evidence for formulating effective measures for antibiotic management.

## Methods

### Study Population and Data Collection

A nationally cross-sectional study on the online antibiotic purchase behavior was designed and conducted among Chinese residents. The data was collected as a part of the community survey module of the undergraduate students of Hainan Medical College using a web-based questionnaire. From January 20 and February 28, 2019, participants were recruited from these student’s native place communities, using the convenience sampling method. The inclusion criteria for the subjects were: 1) 18 years and older 2) those who could read Chinese and 3) able to use smartphone. We also excluded those with severe mental/cognitive problems.

The minimum number of residents required to estimate the prevalence of antibiotic purchase online was calculated. We used the formula for calculating the sample size of the cross-sectional study: *n = z* ^*2*^ *p*(*1-p*)*/d* ^*2*^, where *n* is the sample size, *p* is the proportion of purchasing antibiotic online, *z* is the normal deviation (1.96) and *d* is the margin of error (5%*p*). There was no previous study on the prevalence of antibiotic purchase online, therefore, we replaced the proportion with that of purchasing medicine online to calculate the sample size. Previous research on medicine purchase online had reported a prevalence of 0.45–12.80% (Atkinson et al., 2009; Bowman et al., 2020). And thus, we assumed that 5% of residents purchased antibiotics online (*p* = 5%) and set the confidence interval (*CI*) at 95%. The sample size calculated was 29,196. Furthermore, a study on the sample size estimation showed that the research design efficiency value should be considered when calculating the sample size in a sampling survey. The efficiency value of research design is the ratio of the variance of the planned sampling method estimator to the variance of the simple random sampling method estimator, when the survey unit is the same for the same target quantity (Lv and Feng, 2016). The required sample size can be obtained by multiplying the sample size calculated using the formula by the research design efficiency value. In our study, we assumed the research design efficiency value of the convenience sampling was three, and therefore, the required sample size was 87,588 (29,196*3). According to a web-based survey conducted in China, the response rate of residents was 61% (Li et al., 2020). Considering this response rate, an ideal sample size of 143,587 was needed in our study.

With the help of local community workers, these students who received unified training, identified the eligible residents and sent the questionnaire link to them through social networks. After clicking the link, respondents will first access the informed consent page, which includes the purpose of the investigation, the institution conducting the investigation, assurance of anonymity, and a question: “Do you agree to participate in this survey (Yes/No).” Only respondents who chose “Yes” were directed to the questionnaire page. Considering that some respondents are not familiar with antibiotics, we set a screening item at the beginning of the questionnaire: “Do you know the term of “antibiotic” or drugs like penicillin, amoxicillin, cephalosporin, and roxithromycin? (Yes/No).” If the screening item was answered with “No,” the survey ended, and if the screening item was answered with “Yes,” the survey continued. In addition, we set up three quality control questions at different places in the questionnaire to identify the respondents who filled the questionnaire at random. The three questions were “Where is the capital of China?”, “What is 7 minus 2?”, and “What is 1 plus 3?”. Each question had 4 alternative answers, of which only one was correct, and the correct alternatives for the 3 questions were inconsistent. After answering all the questions, respondents were directed to submit the questionnaire. The questionnaire system would mark the questionnaire as invalid if any of the three answers of the quality control questions was incorrect. Additionally, to prevent multiple submissions, each mobile phone or computer was eligible to answer only once. The answers to all respondent’s questionnaires were automatically entered into a data file and checked by two independent researchers. Respondent’s information was completely confidential and recorded anonymously. This study is a joint research project of Tongji Medical College, Huazhong University of Science and Technology and Hainan Medical College. The former institution was responsible for the study design and the latter was responsible for the data collection. The Medical Research Ethics Committee of Tongji Medical College, Huazhong University of Science and Technology approved the study [no. IEC (S175)] on March 1, 2018.

### Measures

#### Antibiotic Purchase Online

Our primary outcome variable was the purchase of antibiotics online. Respondents were asked whether they bought any antibiotics online in the past 3 months. We defined respondents as purchasers of antibiotics online if they answered yes to the question.

#### Sociodemographic and Health-Related Variables

These variables included age (18–44, 45–64, or ≥65 years of age), gender (male or female), ethnicity (Han or minority), geographic location (east, central, or west), residence (urban or rural), education level (high school or below, associate or bachelor degree, or graduate degree or higher), marital status (never married, married and living with a partner, or divorced or widowed), employment status (employed or not employed), self-reported economic status (excellent, good, fair, poor, or terrible), health insurance status (insured or uninsured), whether suffering from physician-diagnosed chronic diseases (yes or no), and whether searching for health information online in the past 3 months (yes or no).

### Data Analysis

To account for the oversampling and the nonresponse, we used post-stratification weights, which corrected the distribution of respondents to match the known distribution of the Chinese population on age group, gender, residence, and geographic location (National Bureau of Statistics, 2010). We calculated the weighted prevalence of purchasing antibiotics online to obtain a nationally representative estimate. Descriptive analyses were reported as frequency and percentage. Chi-square tests were used to compare characteristics between those who purchased antibiotics online in the past 3 months and those who did not. We did univariable and multivariable logistic regression analyses to identify factors associated with antibiotic purchase online. The likelihood ration goodness-of-fit test was used for the multiple logistic regression. PROC SURVEY procedures were used in SAS version 9.4 (SAS Institute Inc.) to incorporate sampling weights into inferential statistics. All comparisons were two-tailed. We considered differences to be statistically significant if *p* was less than 0.05.

## Results

### Characteristics of Study Respondents

During the study period, we distributed 171,335 questionnaire links, and 107,650 participants from 31 Chinese mainland provinces (municipalities, autonomous regions) completed the online questionnaire, with a response rate of 62.83%. Of them, 6530 who failed to pass the quality control questions were excluded. Finally, 101,120 respondents were included in the analysis. The mean age of the respondents was 30.43 (standard deviation: 13.12) years. Over half were female (58%), unmarried (63%), and living in urban area (60%). As for geographic location, 55, 22, and 23% lived in the eastern, central, and western China, respectively. Furthermore, 68% had an associate or bachelor degree, 91% had health insurance, 50% was employed, as well as 59% reported that their economic status was fair. Nearly one seventh of them (14%) suffered from physician-diagnosed chronic diseases, and 42% had searched for health information online in the past 3 months. Respondent’s characteristics are detailed in Table 1.

**TABLE 1:** Resident’s characteristics and their associations with antibiotic purchase online in the past 3 months.

Characteristic	Total No. (%)	Antibiotic purchase online		*p* Value
		Unweighted frequency and percent No. (%)	Weighted percent^a^ % (95% CI)	
Overall	101120	3318 (3.28)	3.71 (3.53–3.88)	NA
Age (years)				<0.0001
18–44	82394 (81.48)	2654 (3.22)	3.34 (3.20–3.47)	
45–64	16914 (16.73)	539 (3.19)	3.29 (2.99–3.58)	
≥65	1812 (1.79)	125 (6.90)	6.93 (5.73–8.14)	
Gender				<0.0001
Male	42930 (42.45)	1710 (3.98)	4.31 (4.04–4.57)	
Female	58190 (57.55)	1608 (2.76)	3.09 (2.86–3.32)	
Ethnicity				0.0201
Han	90876 (89.87)	2954 (3.25)	3.63 (3.45–3.81)	
Minority	10244 (10.13)	364 (3.55)	4.44 (3.78–5.09)	
Geographic location				0.3213
East	55797 (55.18)	1903 (3.41)	3.82 (3.59–4.06)	
Central	21812 (21.57)	669 (3.07)	3.50 (3.15–3.85)	
West	23511 (23.25)	746 (3.17)	3.77 (3.41–4.12)	
Residence				0.0008
Urban	61036 (60.36)	1951 (3.20)	3.41 (3.21–3.61)	
Rural	40084 (39.64)	1367 (3.41)	4.02 (3.73–4.32)	
Education level				<0.0001
High school or below	27908 (27.60)	682 (2.44)	2.66 (2.38–2.93)	
Associate or bachelor degree	69035 (68.27)	2364 (3.42)	4.01 (3.79–4.23)	
Graduate degree or higher	4177 (4.13)	272 (6.51)	8.07 (6.72–9.41)	
Marital status				0.1394
Never married	63645 (62.94)	2034 (3.20)	3.61 (3.41–3.81)	
Married and living with a partner	35628 (35.23)	1193 (3.35)	3.71 (3.42–3.99)	
Divorced or widowed	1847 (1.83)	91 (4.93)	5.09 (3.62–6.55)	
Employment				<0.0001
Not employed	50926 (50.36)	1578 (3.10)	3.32 (3.10–3.54)	
Employed	50194 (49.64)	1740 (3.47)	4.05 (3.78–4.31)	
Self-reported economic status				<0.0001
Excellent	5626 (5.56)	417 (7.41)	7.70 (6.68–8.72)	
Good	11207 (11.08)	526 (4.69)	5.45 (4.79–6.11)	
Fair	60064 (59.40)	1592 (2.65)	2.85 (2.66–3.03)	
Poor	16233 (16.05)	468 (2.88)	3.00 (2.65–3.36)	
Terrible	7990 (7.90)	315 (3.94)	5.10 (4.28–5.91)	
Health insurance				0.5833
Uninsured	8749 (8.65)	283 (3.23)	3.88 (3.24–4.52)	
Insured	92371 (91.35)	3035 (3.29)	3.69 (3.51–3.88)	
Chronic disease				<0.0001
No	86834 (85.87)	2610 (3.01)	3.25 (3.08–3.41)	
Yes	14286 (14.13)	708 (4.96)	5.62 (5.03–6.22)	
Searching for health information online				<0.0001
No	58650 (58.00)	998 (1.70)	2.22 (2.02–2.43)	
Yes	42470 (42.00)	2320 (5.46)	6.09 (5.77–6.41)	

CI, confidence interval; NA, not applicable.

a Estimates are weighted to be nationally representative.

### Prevalence of Antibiotic Purchase Online in the Past Three Months

The overall weighted prevalence of antibiotic purchase online in the past 3 months was 3.71% (95% confidence interval [95% CI], 3.53–3.88%). Additionally, the weighted prevalence was more than 6% (1.5 times of the overall weighted prevalence) among those who were aged 65 years or older, and who had a graduate degree or higher education level, reported an excellent economic status, or searched for health information online. Whereas, there was no difference in the prevalence among residents with different marital or insurance status, as well as among those who lived in different regions of China. Table 1 presents the results of frequency and prevalence of antibiotic purchase online in the past 3 months among residents according to their characteristics. Supplementary Figure S1 shows the prevalence of online antibiotic purchases in each province of China.

### Factors Associated With Antibiotic Purchase Online in the Past Three Months

The crude and adjusted OR (aOR) for factors associated with antibiotic purchase online are shown in Table 2. After controlling for confounders, those aged 65 years or older were more likely to purchase antibiotics online than those aged 18–44 years (aOR, 2.18; 95% CI, 1.74–2.74). Being a female (aOR, 0.69; 95% CI, 0.62–0.77) was associated with reduced likelihood to purchase antibiotics online. Regarding self-reported economic status, residents with a good, fair, poor, or terrible economic status were all less likely to purchase antibiotics online compared with those reporting an excellent economic status (the values of four aOR and their 95%CIs were all less than one).

**TABLE 2:** Univariable and multivariable logistic regression of factors associated with antibiotic purchase online in the past 3 months.

Characteristic	Crude OR (95% CI)	*p* Value	Adjusted OR (95% CI)^a^	*p* Value
Age (years)				
18–44	1.00		1.00	
45–64	0.98 (0.89–1.09)	0.7630	1.09 (0.95–1.25)	0.2067
≥65	2.16 (1.78–2.61)	<0.0001	2.18 (1.74–2.74)	<0.0001
Gender				
Male	1.00		1.00	
Female	0.71 (0.64–0.78)	<0.0001	0.69 (0.62–0.77)	<0.0001
Ethnicity				
Han	1.00		1.00	
Minority	1.23 (1.05–1.45)	0.0117	1.15 (0.97–1.37)	0.1023
Geographic location				
East	1.00		1.00	
Central	0.91 (0.81–1.03)	0.1425	0.93 (0.83–1.06)	0.2712
West	0.98 (0.88–1.11)	0.7909	0.96 (0.85–1.08)	0.4653
Residence				
Urban	1.00		1.00	
Rural	1.19 (1.08–1.31)	0.0006	1.43 (1.29–1.58)	<0.0001
Education level				
High school or below	1.00		1.00	
Associate or bachelor degree	1.53 (1.36–1.73)	<0.0001	1.51 (1.31–1.74)	<0.0001
Graduate degree or higher	3.22 (2.61–3.97)	<0.0001	2.11 (1.66–2.67)	<0.0001
Marital status				
Never married	1.00		1.00	
Married and living with a partner	1.03 (0.93–1.14)	0.5922	0.91 (0.77–1.06)	0.2277
Divorced or widowed	1.43 (1.05–1.95)	0.0233	0.97 (0.67–1.40)	0.8763
Employment				
Not employed	1.00		1.00	
Employed	1.23 (1.11–1.35)	<0.0001	1.06 (0.94–1.20)	0.3554
Self-reported economic status				
Excellent	1.00		1.00	
Good	0.69 (0.57–0.84)	0.0002	0.76 (0.62–0.93)	0.0084
Fair	0.35 (0.30–0.41)	<0.0001	0.45 (0.37–0.53)	<0.0001
Poor	0.37 (0.31–0.45)	<0.0001	0.44 (0.36–0.54)	<0.0001
Terrible	0.64 (0.52–0.80)	<0.0001	0.70 (0.55–0.90)	0.0057
Health insurance				
Uninsured	1.00		1.00	
Insured	0.95 (0.79–1.14)	0.5756	0.90 (0.75–1.10)	0.2997
Chronic disease				
No	1.00		1.00	
Yes	1.77 (1.57–2.01)	<0.0001	1.44 (1.27–1.63)	<0.0001
Searching for health information online				
No	1.00		1.00	
Yes	2.85 (2.56–3.18)	<0.0001	3.10 (2.79–3.46)	<0.0001

CI, confidence interval; OR, odds ratio.

a Adjusted for the other eleven variables.

Additionally, residents who lived in rural areas (aOR, 1.43; 95% CI, 1.29–1.58), or suffered from chronic diseases (aOR, 1.44; 95% CI, 1.27–1.63) had higher odds of purchasing antibiotics online. Compared with those having a high school or lower degree, residents who had an associate or bachelor degree (aOR, 1.51; 95% CI, 1.31–1.74) as well as a graduate or higher degree (aOR, 2.11; 95% CI, 1.66–2.67) were more likely to purchase antibiotics from online pharmacies. In the past 3 months, those had searched for health information on the internet were more than three times (aOR, 3.10; 95% CI, 2.79–3.46) more likely to purchase antibiotics online.

## Discussion

Our study showed that 3.71% of Chinese residents had purchased antibiotics on the internet in the past 3 months. Currently, few study on online antibiotic purchase behavior of residents has been conducted in other countries, but studies on prescription medicine purchase online have been carried out. Relevant studies showed that 0.45 and 5.00% of residents have purchased prescription medicines on the internet in Malta and the United States in the past year, respectively (Baker et al., 2003; Bowman et al., 2020). Though the overall prevalence of 3.71% is relatively low in our study, this amount represents numerous people having purchased antibiotics online throughout China. This study did not investigate whether the respondents purchased antibiotics from online pharmacies with a prescription or not, but previous studies have reported that lots of Chinese people were accustomed to purchase antibiotics without a prescription. A study revealed that 50.6% of Chinese residents had purchased antibiotics without a prescription in retail pharmacies in the past year (Jiang et al., 2021). Furthermore, there is a large proportion of online pharmacies selling antibiotics without a prescription in China (Gong et al., 2020). These suggest that the online antibiotic purchase behavior of residents should not be neglected and enhanced supervision of internet sales of antibiotics is needed.

The current study found that the female was less likely to buy antibiotics on the internet, which may be due to that they were more cautious about medicine purchase online. A study on the online medicine purchase behavior of Chinese residents reported that women were less inclined to buy medicines in online pharmacies due to their cautiousness (Jia et al., 2018). Additionally, studies showed that convenience was an important reason for residents to purchase medicines online (Fittler et al., 2018; Bowman et al., 2020). This may also be the reason for the elderly and rural resident’s preference for purchasing antibiotics online. In China, the electronic commerce has developed rapidly and been widely popularized (Liang, 2017). The inconvenience in movement of the elderly and the lack of medical resources in rural areas may encourage the elderly and rural residents to purchase antibiotics in online pharmacies. Overall, online pharmacies might provide convenience for residents to obtain antibiotics. But at the same time, we should note that antibiotic can easily be used irrationally, especially in China, this issue is extremely serious (Fang, 2014; Lv et al., 2014; Yu et al., 2014). Therefore, how to ensure the convenience of online purchases of antibiotics, and the standard management of antibiotics at the same time, is worthy of discussion by relevant ministries. Furthermore, due to the physiological features of the elderly, the consequence of irrational use of antibiotics is more severely for them, which should be paid enough attention (Pea, 2018).

In our study, residents with higher education level were more prone to purchase antibiotics online. This is consistent with the result of studies on factors related to self-medication with antibiotics, which found that higher education level was associated with irrational use of antibiotics (Hounsa et al., 2010; Elong Ekambi et al., 2019). People with higher levels of education believe that they have a certain “medical savvy,", and often overestimate their understanding of antibiotics. Therefore, they may be more likely to feel confident in diagnosing an illness and, consequently, in self-prescribing an antibiotic therapy (Grosso et al., 2012). Notably, residents who suffered from chronic diseases or reported an excellent economic status were associated with greater likelihood of buying antibiotics on the internet. This can be explained by that these people may pay more attention to their health status, and when they suffer from minor illness, they would likely to take antibiotics to relieve symptoms. In addition, many residents with chronic diseases usually feel stigma (Sarfo et al., 2017; Liu et al., 2020; Himmelstein and Puhl, 2021). Face-to-face communication with the pharmacist can be avoided by online purchases of antibiotics, which can reduce their psychological stress.

In the past 3 months, nearly half of the residents had searched for health information on the internet, and they were more likely to purchase antibiotics online. This may be due to that they usually chose to seek information online to self-diagnose and self-medication when they suffered from illness. Information obtained online and internet usage habits may encourage them to purchase medicines online. It is very important to ensure that the internet provides correct health information and medication knowledge in the information age.

### Strengths and Limitations

To our knowledge, this is the first study to explore the prevalence of antibiotic purchase online among residents and identify its associated factors. We considered that this study will help the researchers and policymakers worldwide understand the online antibiotic purchase behavior of residents, as well as provide evidence for antibiotic stewardship. This study has several limitations. First, this study was conducted by using a convenience sampling method and web-based survey. These may affect the sample’s representativeness. However, the survey was conducted by trained investigators in urban and rural communities all over the country, the sample size was large, and post-stratification weights were used to correct the distribution of the respondents, which can make the results more nationally representative. Second, we investigated the online antibiotic purchase behavior in the past 3 months, and therefore, recall bias may exist. Third, we did not collect the information on types of antibiotics purchased online, medical conditions that required antibiotics, whether residents purchased antibiotics with a prescription or not, and other possible factors influencing the purchase behavior, which limited our more comprehensive understanding of the online antibiotic purchase behavior of residents.

### Practical Implications

Our study investigated the online antibiotic purchase behavior of residents, which could provide evidence for researchers and policymakers worldwide to understand this behavior. Additionally, the study results showed that the prevalence of antibiotic purchase online was relatively high among Chinese residents. This implied that, on the one hand, the regulation of antibiotic sales online should be strengthened; on the other hand, there is a need to improve public education on antibiotic purchase online, so as to promote purchases of antibiotics with a prescription and rational use of antibiotics.

### Future Researches

As a new channel to obtain antibiotics, online antibiotic purchase has rarely been studied. Therefore, it is necessary to pay attention to this purchase behavior, and carry out more relevant researches. For the future studies, more detailed information of antibiotic purchase online should be collected, such as types of antibiotics purchased and whether residents purchased antibiotics with a prescription or not, so as to provide a more comprehensive reference for antibiotic stewardship. In addition, antibiotic is an important kind of medicines for the treatment of acute bacterial diseases. Online sales of antibiotics have improved the accessibility of these medicines among residents. However, considering that antibiotics should be used accurately and timely for patients when needed, whether online purchases of antibiotics will result in delay in treatment is a matter of concern. Extensive researches and discussions should be conducted to reveal the advantages and disadvantages of online sales of antibiotics.

## Conclusion

Our study found that numerous Chinese residents had purchased antibiotics on the internet. Age, gender, residence, education level, economic status, suffering from chronic diseases, and searching for health information on the internet were all significantly associated with the purchase behavior. Therefore, it is necessary to strengthen the regulation of antibiotic sales on the internet, and improve public education on antibiotic purchase online. In the future studies, more comprehensive information on antibiotic purchase online should be collected, as well as the advantages and disadvantages of online sales of antibiotics should be investigated.

## Data Availability

The raw data supporting the conclusions of this article will be made available from the corresponding author on reasonable request.
