# Supplementary material for: Prevalence of Antibiotic Purchase Online and Associated Factors Among Chinese Residents: A Nationwide Community Survey of 2019
Source: Front Pharmacol. 2021 Nov 3;12:761086. doi: 10.3389/fphar.2021.761086 (PMC8595837; doi:10.3389/fphar.2021.761086)
Supplement: Supplementary file 1 [file Image1.PDF]

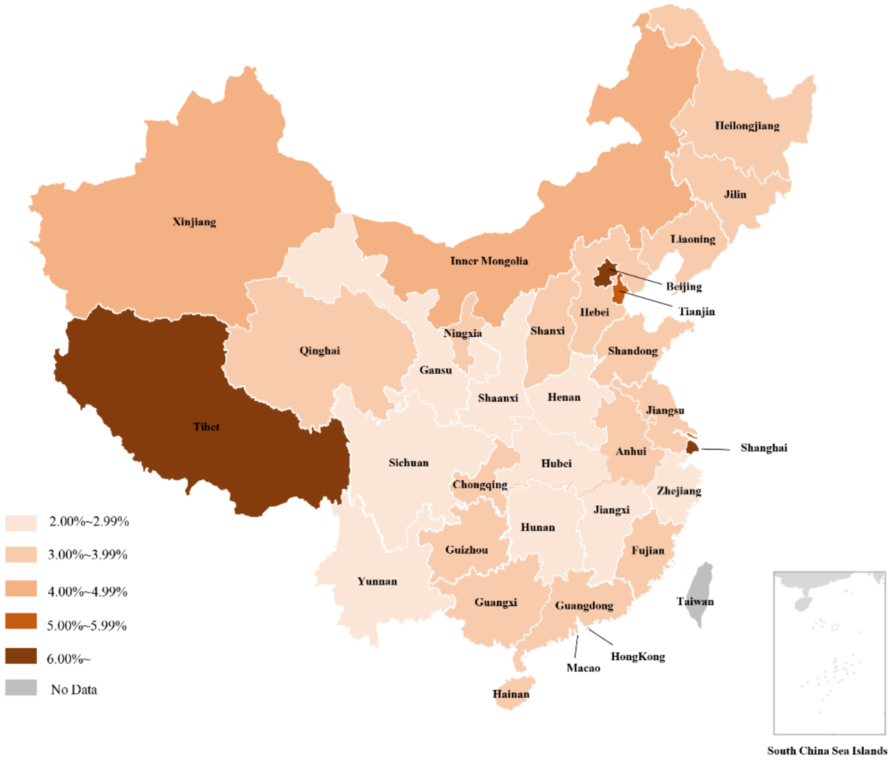

Appendix Fig. 1 The prevalence of online antibiotic purchases in each province of China

Figure legend: The sample population of this study was from all over China except Taiwan, Hong Kong, and Macau.
